# Aneurism of the Arch of the Aorta, Fatal from Rupture into the Anterior Mediastinum

**Published:** 1830-08

**Authors:** Alex. Thomson


### ANEURISM OF THE AORTA.

*Aneurism of the Arch of the Aorta, fatal from Rupture into the anterior Mediastinum.* By Alex. Thomson, m.b.

Eliza Blackburn, aged 28, well made, upwards of six feet in height, married, and the mother of five children, the last of whom was born about four years back. She was hasty and passionate, took powerful exercise, and danced often to excess, when in tolerable health. About four years ago she was afflicted with hysteria, which attacked her from time to time for the space of twelve months, and then left her. During these last four years, she has done little or no work, on account of constant and overpowering debility, brought on, or rather materially increased, even by slight exertion. From childhood she was very subject to colds, which always settled in the throat, and terminated in what is commonly called ulcerated sore throat. The last time she was affected in this manner was about four years ago, when the tonsils, being so swollen as to prevent deglutition, were punctured by her medical attendant, Mr. Low. From this period she has had no more similar attacks. She was never affected with cough until last autumn, when, after catching cold, she was seized with a loud, coarse, hollow cough, which continued for two or three months before she began to expectorate. From this period to that of her death, she continued to expectorate a viscid frothy matter, resembling the white of an egg, occasionally streaked with blood, whenever the cough was more than usually violent. Her cough did not come on in long paroxysms. She complained, as much as four years back, of pains in both shoulders, but more particularly in the left shoulderblade, which, together with debility, prevented her from working, as both were brought on by exertion. For twelve months before her death, she had very severe pains in the right arm, which were constant, but increased by a change in the weather; latterly this arm used to swell considerably.

During the winter before last, for the first time she was seized with a violent pain in the chest, at about the middle of the sternum, so violent as to prevent her lying down, and accompanied by intense pain of the right arm. She never was heard by her mother, her constant companion, to complain of beating or of a sense of burning in the chest. For this pain she was attended by Mr. Cope, of Cleveland street, who soon relieved her, and enabled her to go about her usual avocations. She felt no more of the pain in the chest until last Christmas, when it returned with increased violence, and, although materially relieved at that time by a large blister, continued almost unabated till her death. She also suffered from intense pain of the left side, in the region of the heart, and was never able to lie upon that side. She slept very heavily during the night, and almost invariably for two or three hours after dinner; was much troubled with horrible dreams, and frequently awakened with a start. For some nights previous to her death, she was exceedingly restless. She has never been able to lie down since last Christmas, but has been obliged to be supported with pillows nearly in a sitting posture. Her appetite was never very good, and her dinner invariably rendered her very uneasy, making her feel (to use her own expression,) "as though she was young with child." She was latterly very anxious and unhappy in her mind. Till last Christmas she was tolerably regular in her bowels, but since that period she has been almost invariably constipated. The stools have been indurated, scybalous, and dark coloured; the urine abundant, and passed with ease; the catamenial discharge regular in its recurrence at the end of three weeks, but always (at least in the opinion of the mother) too copious. It occurred a few days before her death, and was followed by material relief of her most painful symptoms. She had complained, since Christmas last, of a pain in the small of her back. About a fortnight since, the veins of the left side of her neck were enormously swollen, and remained so, producing considerable pain. She frequently felt very faint, particularly on the evening of the day previous to that on which she died, although in the morning she had been in very good spirits.

On the evening of her death, she had her feet bathed in hot water, by which she had often found her chest relieved, and was put to bed as usual at half-past ten, where, propped by pillows, she composed herself to sleep. At twelve P.M. she roused up the persons of the house, and asked them to send for the doctor. In less than half an hour from this period, she clasped her hands, and, without a sigh or a groan, leant upon her right side, and died.

I must not omit to observe, that she was never troubled with difficulty of breathing. I saw her but once previous to her death, and that was at her last visit to the dispensary, at which she had been treated for chronic bronchitis. No suspicion had been entertained of any affection of the aorta. Struck with her appearance, and an unusual pulsation between the sterno-clavicular ends of the sterno-mastoid muscles, and suspecting disease of the heart, I applied the stethoscope to her chest, over the upper part of the sternum, when I heard a sound as of water poured from a wide-mouthed bottle into a basin already containing water. I was, therefore, requested to call upon her, and examine her more carefully at my leisure; a request which I was prevented complying with, by hearing next morning but one of her death.

*Morbid appearances.* On opening the thorax, the margins of the lungs were remarkably healthy, although attached by recent adhesions to the mediastinum, which evidently contained a tumor separating its layers, and so closely adherent to the triangular bone of the sternum, as necessarily to be penetrated by an attempt to remove it by separating it from the periosteum. It is worthy of remark that the first incision into the superficial veins of the neck was followed by a jet, or rather continuous fountain of blood, which, although no part of the neck or body was higher than the part cut, rose from the orifice of the incision to the height of about half an inch, and continued to flow with rapidity for the space of two or three minutes. It was difficult to account for such a flow of blood from a body in which all the functions of life had been suspended for upwards of twenty-four hours, in which also the joints were rigid and the whole frame cold; and those present were so much struck as for a moment to suspend the examination. The neck had been purposely elevated by a block, in order to permit of an examination of the larynx and trachea, and therefore the lower part of the neck, in which the incision was made, was at that time higher than any other part of the body. Gravity, therefore, was not the agent by which the still fluid blood was put in motion; nor could atmospheric pressure exert any influence, inasmuch as, the vein being opened, the stream of blood was equally acted upon by the atmosphere as the coats of the vessels themselves. It is true, indeed, that all the veins of the neck, as had been remarked previously to the commencement of the examination, were remarkably swollen or distended; and it appears probable that this turgescence of the veins aided the elastic or resilient power of the coats of the veins, (for it appears that we must grant the existence of such a power, and from this observation, that it continues for some time, even twenty-four hours, after death,) in forming this fountain.

Both pleural sacs contained a small quantity of sanious serum, and the vessels of the costal and pulmonary pleuræ of both sides were considerably injected. There were, however, no adhesions besides those already mentioned, and no affection of the substance of the lungs, except venous congestion and a highly-injected state of the mucous membrane of the bronchial tubes. There were not, however, any ulcerations noticed in these tubes, nor any remarkable thickening of their parietes, nor any cavities or tubercles, or infiltration into the parenchyma of the lungs.

The tumor in the centre of the anterior mediastinum, was closely attached by dense cellular substance to the whole of the surface of the triangular bone of the sternum, and over a space of about the magnitude of a shilling to the contiguous part of the second bone of the sternum. The sac of the tumor was in close and intimate adhesion with the periosteum of the bone, so close that it could with difficulty be separated. The sac of the tumor was at this part fibro-cartilaginous, and offered considerable resistance to the knife. Over by far the greater portion of the bone to which the sac adhered, beginning from its lowest point and proceeding upwards to the right sterno-clavicular junction, the periosteum, though still adherent by cellular tissue and small vessels, could be torn from the bone by the slightest touch.

*The large sac,* into which an incision was made in order to separate the sternum, contained one beer-pint of dark, venous looking, very fluid blood. After standing for some time, it began to coagulate. The tumor extended upwards till it came in contact with the thyroid glands laterally, on either side, as far as the terminations of the cartilages of the ribs, and on the right side, towards the middle, to some little distance beyond them, inferiorly to the diaphragm. It occupied the lower anterior half of the neck, came anteriorly in contact with, and adhered firmly to, the major part of the triangular bone of the sternum; occupied the whole of the anterior mediastinum, separating its layers, and receiving them as coats for itself; extended on the right side to the inner margin of the common carotid, with the coat of which it was in adhesion as far down as the aorta; lower down traversed the margin of the origin of the lung; then, having its boundary turned towards the median line, crossed with it the right phrenic nerve, and, passing obliquely downwards and forwards, arrived at the tendinous part of the diaphragm, with the whole of the anterior and lateral margins of which it was in contact. Its boundary on the left side, passing obliquely upwards, backwards, and inwards, crossed the left phrenic nerve at about the centre of the origin of the left lung; proceeded upwards, backwards, and inwards again to the margin of the trachea, along which it ran till it again came in contact with the thyroid body. On either side of the tumor, the layers of the mediastinum were intensely inflamed, and in some parts attached to the adjacent surface of the lungs by adhesions, themselves minutely traversed with longitudinal vessels, loaded with red blood. At that part of the mediastinum which is reflected from the diaphragm, there was a large mass of dark, black coagulum, occupying the intervening cellular tissue, and elevating the mediastino-pericardial pleura, over a space of about four square inches, from the diaphragm upwards. There were, moreover, several patches of effused blood between the mediastinal pleura and the parietes of the tumor, where the vessels of the former were most remarkably injected.

This tumor, when more narrowly examined, proved to be a vast aneurism, proceeding from that part of the anterior superior paries of the aorta where that vessel, escaping from the pericardium, begins to form its arch. The aperture by which the vessel opened into the aneurismal sac was of a bluntly rhomboidal form, the greater axis of the rhomboid being vertical. At the superior point of the longer axis of this aperture was seen the orifice of the innominata, and a little to the left and below it was that of the subclavian. This was manifestly, in the first instance, an aneurism by dilatation, because the sac of the aneurism was formed of membranes in all respects analogous to the coats of the aorta, and particularly to the appearance of these coats when they are simply dilated. The internal or lining membrane of the sac was not only continuous with that of the vessel, but smooth and polished. Its continuity with the lining membrane of the vessel was easily traced in one spot, where accidental traction had torn the margin of the orifice. Exterior to the lining membrane was a fibrous, elastic, yellow or buff coloured membrane, thinner it is true in some parts, but in all other respects similar to the lining membrane of the aorta. Between the inner and middle coats of the sac were numerous irregular semi-cartilaginous plates, similar to those which are found in the same position in the aorta. Upon an incision being made across the margin or edge of the orifice leading from the vessel, where the membranes were reflected backwards, both the internal and middle membranes of the aorta could be traced curving round at the margin to form the parietes of the sac.

In this section it appeared, at that part where the dilated vessel was in contact and attachment with the undilated, that the coats of the tumor were three in number: an internal, reddish, translucent coat, polished on its unattached surface; a middle, opaque, yellow, fibrous coat; and an external one, consisting of cellular substance. As soon as the parietes were cut across, after they had come in contact with the pericardium or mediastinal pleura, a fourth coat, in addition to those already mentioned, could be traced. Again, along the middle of the sac, when laid open, might be seen running transversely to the perpendicular axis of the tumor, beneath the smooth and polished lining membrane, the zigzag ruptured margins of the yellow, fibrous membrane. On either side, to the right and left of the median line, the zigzag margin of the one side corresponded to that of the opposite side in its irregularities. It appeared that when this middle membrane had given way, the inner one had only been dilated, since the polished smooth membrane extended for an inch and a half all round the sac beyond the margin of the yellow one. Finally, this polished membrane having given way at the most depending part of the tumor, and at the point most remote from the orifice of the sac, had allowed of the blood being effused into the cellular substance of the mediastinum, so as to separate that membrane from its connexions with the pericardium. The lining membrane of that part of the sac, consisting of four coats, was in different parts covered with inflamed and ecchymosed patches, on several of which small coagula had been formed and become attached. The lining membrane of the aorta itself, opposite to the dilatation, was considerably thickened, reddish, and elevated by semi-cartilaginous plates or depositions.

*The trachea* and *larger bronchial tubes* were considerably inflamed.

*The pericardium* contained about half a pint of glairy, rather turbid, pinkish fluid, of which some, when heated, coagulated, and the whole, when it had stood for half an hour, became cupped like blood under ordinary circumstances, separating into coagulum and serum; yet no blood had been effused into the pericardium, nor had the sac of the aneurism any connexion whatever with that of the pericardium. The internal surface of the pericardium was densely covered with vessels containing red blood, and elevated by numerous petechial spots along that tract, corresponding to the margin of the coagulum effused by the rupture of the aneurismal sac between the pericardium and the mediastinum. The cardial part of the pericardium had its vessels highly injected with red blood, and in some parts was spotted with stellular blushes of inflammation. The heart itself was smaller than natural, empty and contracted, loaded along the course of the coronary vessels with fat, softer than usual in its substance; and the parietes of both ventricles unusually injected with red blood. All the valves but those of the aorta, which had small semi-cartilaginous depositions along their attached margins, were healthy. The lining membrane of the ascending aorta was much thickened, unusually red, and elevated by numerous, irregularly-shaped, semi-cartilaginous plates.

Abdominal viscera, and the contents of the cranium, of healthy appearance.

*Remarks.* This case is interesting in several points of view. The aneurism crept on without giving any alarming indications to the patient, and, though completed by successive ruptures, still produced, till latterly, but slight inconvenience at the various periods of these accidents.

The first time she experienced pain in the thorax was about one and a half years before her death. It is more than probable that the laceration of the fibrous coat of the tumor took place about this time; for the pain at about the middle of the sternum was so intense as to prevent her lying down or obtaining rest. Yet she shortly recovered from this pain under the care of Mr. Cope, and, with the exception of debility, was tolerably well in health. A year elapsed before she again experienced inconvenience, which appears to have begun under the influence of mental agitation. From this time the unceasing pain in the region of the heart, accompanied by the same anguish at about the middle of the sternum, the dreamy and uncertain rest at night, disturbed by the startings, the inability to recline upon the left side, and to lie down flat at all, might, one would think upon reflection, have induced her medical attendants to refer the complaint to the heart. The debility itself, a sign, when unaccompanied by emaciation, as in this case, of disordered circulation, might have strengthened their suspicion, but they referred all these indications to angina pectoris, and they feared the stethoscope, because they knew not how to use it, and had never cared to investigate its powers. That she had bronchitis is true, from the evidence of the morbid examination, but that such bronchitis is the ordinary accompaniment of diseased heart or appendages, ought not to have been forgotten by the practitioner, had he carefully considered or investigated the previous history of the case.

These remarks are the more useful because, on this occasion, the physician who attended her in her last illness, derided publicly and before his pupils the use of the stethoscope on this very occasion; and, though one of the high priests of opposition to the introduction of this invaluable aid to practice, he did, upon viewing the dissection, and remembering the result of my last hurried examination of the chest, condescendingly admit that this tube might hereafter, but he feared at some distant period, be of use to the practitioner. His fearless denial of any other existing disease but bronchitis, was a striking warning to his pupils, and may well afford the same lesson to those who consider nothing good, but what themselves have learned to value in the early periods of their career.

It is curious that the blood of the aneurism remained quite fluid until drawn from the body twenty-four hours after death, when it suddenly coagulated; and this fact is the more remarkable from its being arterial blood which had remained in contact so long with ragged cellular tissue. If death of the blood causes its coagulation, in what state was this blood during the twenty-four hours? If arterial blood coagulate immediately upon coming in contact with cellular tissue, why did it in this case not coagulate under the favorable circumstances in which it was placed in the aneurismal sac.

The preparation of the sac of the tumor may be seen in the museum of the University of London.

